# ERβ localization influenced outcomes of EGFR-TKI treatment in NSCLC patients with EGFR mutations

**DOI:** 10.1038/srep11392

**Published:** 2015-06-22

**Authors:** Zhijie Wang, Zhenxiang Li, Xiaosheng Ding, Zhirong Shen, Zhentao Liu, Tongtong An, Jianchun Duan, Jia Zhong, Meina Wu, Jun Zhao, Minglei Zhuo, Yuyan Wang, Shuhang Wang, Yu Sun, Hua Bai, Jie Wang

**Affiliations:** 1 Department of Thoracic Medical Oncology, Key Laboratory of Carcinogenesis and Translational Research (Ministry of Education), Beijing Cancer Hospital & Beijing Institute for Cancer Research, Beijing, China; 2 Department of Oncology, Aviation General Hospital, Beijing, China; 3 Metabolomics Center, National Institute of Biological Sciences, Beijing, China; 4 Department of Pathology, Key Laboratory of Carcinogenesis and Translational Research (Ministry of Education), Beijing Cancer Hospital & Beijing Institute for Cancer Research, Beijing, China

## Abstract

Effects of estrogen receptorβ (ERβ) localization on epidermal growth factor receptor tyrosine kinase inhibitors (EGFR-TKIs) in advanced non-small cell lung cancer (NSCLC) are unknown. First, we analyzed the relationship between ERβ localization determined by immunohistochemistry and EGFR-TKI outcomes in 184 patients with advanced NSCLC and found that ERβ expression localized in the cytoplasm and/or nucleus. The frequency of cytoplasmic ERβ (c-ERβ) and nuclear ERβ (n-ERβ) co-expression was 12% (22/184). C-ERβ and n-ERβ co-expression was correlated with poor median progression-free survival compared to patients without co-expression. In subsequent *in vitro* experiments, PC9 cells transfected with ERβ isoform1 (ERβ1, strong expression of both c-ERβ and n-ERβ) were more resistant to gefitinib than PC9 cells transfected with ERβ isoform2 or 5 (ERβ2 or ERβ5, strong expression of ERβ in cytoplasm but not nucleus). Resistance was identified due to interactions between ERβ1 and other isoforms, and mediated by activation of non-genomic pathways. Moreover, gefitinib resistance was reversed by a combination treatment with gefitinib and fulvestrant, both in cell lines and in one NSCLC patient. These results suggested that c-ERβ and n-ERβ co-expression was a potential molecular indicator of EGFR-TKI resistance, which might be overcome by combining EGFR-TKI and ER antagonist.

The epidermal growth factor receptor (EGFR) superfamily has been identified in the development of tumor cells and as such has emerged as a therapeutic target. Activation of EGFR sensitizing mutations, such as exon 19del and 21L858R, can significantly predict superior responses to EGFR tyrosine kinase inhibitors (TKIs) in lung adenocarcinoma^1^,^2^,^3^,^4^,^5^. However, primary and acquired resistances to EGFR-TKIs limit the efficacy of these agents. Mechanisms of acquired resistance to TKIs have been discovered, and approximately 70% of patients who fail EGFR-TKI therapy have specific resistance-related gene variants, such as the EGFR T790M mutation and c-MET amplification. However, studies regarding primary resistance to TKIs are limited, which has led to a lack of strategies available to overcome primary resistance.

Estrogen receptors (ERs) are members of the nuclear steroid receptor superfamily. Two forms of ERs have been identified, ERα and ERβ, which are products of two separate genes^6^. The two ERs have different tissue distributions and play inconsistent roles in tumor cell biology. ERβ is commonly overexpressed in human NSCLC cell lines and patients and plays an important role in lung cancer development^7^. Despite the classical model of ERs stimulating transcription of estrogen-responsive genes, non-genomic signaling pathways are also activated by estrogen, including PI3K-AKT-mTOR and MAPK, which induce cancer cell proliferation and apoptosis arrest^8^,^9^. These pathways are considered common downstream signaling mechanisms of EGFR. In several preclinical studies based on lung cancer cell lines and xenografts, EGFR expression was down regulated in response to estrogen and up-regulated in response to ER antagonists (i.e., fulvestrant or tamoxifen) in NSCLC cell lines. Conversely, ERβ protein expression was down-regulated in response to EGF and up-regulated in response to gefitinib (an EGFR-TKI)^10^,^11^. These results indicate an interaction between EGFR and ER-related pathways.

We proposed the hypothesis that ER could induce resistance to EGFR-TKIs in lung cancer and that addition of an ER antagonist could reverse the resistance. However, clinical analysis in a Japanese study showed that strong ERβ expression predicts a better clinical outcome than weak expression in patients with lung adenocarcinoma following EGFR-TKIs therapy^12^. This study did not differentiate between ERβ localization (cytoplasm vs. nuclear), which could alter non-genomic signal pathway and activate and influence clinical outcomes.

To further investigate the impact of ERβ localization on EGFR-TKI efficacy, we analyzed correlations between ERβ localization (cytoplasmic and/or nuclear) and survival after EGFR-TKI therapy in 184 Chinese patients with advanced NSCLC and confirmed the clinical results in lung cancer cell lines. In addition, we first to date illustrated that the interactions between ERβ isoforms were associated with ERβ-mediated resistance to EGFR-TKIs and also explored the rationale for using EGFR-TKIs combined with fulvestrant in EGFR-mutant NSCLC.

## Results

### ERβ expression and correlation with clinical characteristics in patients with advanced NSCLC

A total of 184 patients with stage IV NSCLC treated with EGFR-TKIs were analyzed, and 65 patients were treated as first-line therapy. Clinicopathological characteristics of the patients are summarized in Table 1. Most patients were never/light smokers (122, 66.3%) and had adenocarcinoma (159, 86.4%). A total of 107 patients (58.2%) carried EGFR sensitizing mutations (in exon 19del or 21L858R).

**Figure 1:**
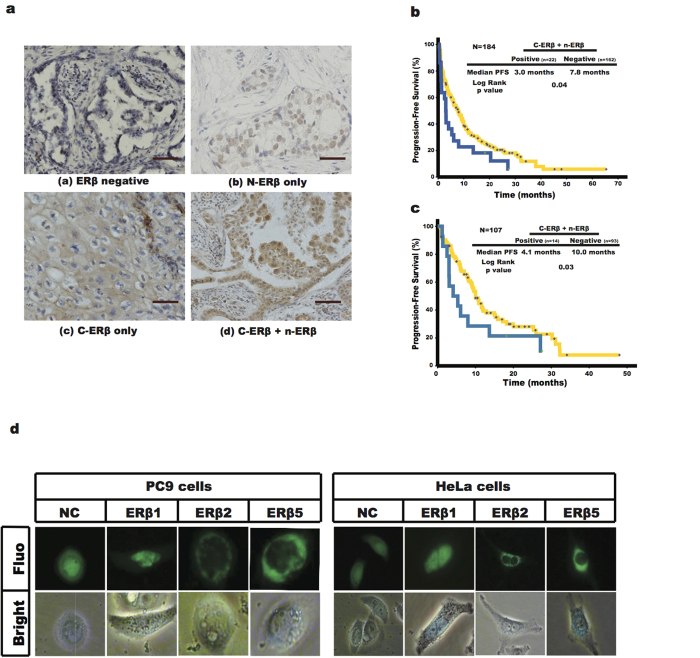
ERβ localization and the correlations with PFS after EGFR-TKI in advanced NSCLC patients. (**A**) Representative Immunohistochemical staining (IHC) of ERβ in tissue specimens obtained from 184 patients with advanced NSCLC. Brown staining indicated ERβ protein. The IHC staining of ERβ protein reflected the different patterns of intracellular localization of ERβ, in which (a) indicated “Negative”, (b) indicated “ERβ positive only in nucleus”, (c) indicated “ERβ positive only in cytoplasm”, and (d) indicated “ERβ positive in both cytoplasm and nucleus”. Scale bars = 200 nm. (**B**) Kaplan-Meier curves illustrated that patients with co-expression of c-ERβ and n-ERβ showed a poorer PFS after EGFR-TKI treatment than those without such expression pattern (*P *= 0.04) in total population (*N *= 184). (**C**) Kaplan-Meier curves showed that co-expression of c-ERβ and n-ERβ predicted inferior PFS after EGFR-TKI treatment compared to those without such expression pattern (*P *= 0.03) in subset with EGFR mutations (*n *= 107). (**D**) indicated the localizations of different ERβ isoforms. The confocal images (upper: fluorescence, lower: bright field) of PC9 and HeLa cells transfected with different ERβ isoform plasmids (NC, ERβ1, ERβ2, and ERβ5) showed that ERβ1 mainly localizes in nuclus contrary to ERβ2 and ERβ5 that mainly localize in cytoplasma.

ERβ expression was positive in 26.6% (49/184) of the patients with different intracellular distribution patterns, including nuclear only (n-ERβ), cytoplasmic and nuclear (c-ERβ and n-ERβ co-expression) and cytoplasmic only (c-ERβ) (22, 22 and 5 patients, respectively) (Fig. 1A).

No significant correlations were observed between ERβ expression and EGFR mutations (*P* = 0.093) or gender (*P* = 0.37). Moreover, neither nuclear nor cytoplasmic expression of ERβ was associated with gender (*P *= 0.586, and *P *= 0.105, respectively) or any other of the clinicopathological characteristics (data not shown).

### C-ERβ and n-ERβ co-expression was correlated with poor survival in patients treated with EGFR-TKI

At the time of data collection (Dec 20, 2013), 148 patients (80.4%) presented with progressive disease (PD) and 95 patients (51.6%) had died. As expected, patients with EGFR sensitizing mutations (*n *= 107) had a significantly superior median PFS compared to patients without such mutations (*n *= 77) (10.0 months vs. 2.8 months, *P* < 0.001). Interestingly, patients with c-ERβ and n-ERβ co-expression had a poorer PFS after EGFR-TKI treatment (*n *= 22, 3.0 months, 95%CI: 2.3 to 3.7 months) than those without co-expression (*n *= 162, 7.8 months, 95%CI: 5.7 to 9.9 months; *P *= 0.04) (Fig. 1B). When categorized by ERβ expression, the median PFS of patients with c-ERβ and n-ERβ co-expression was shorter (3.0 months) compared to patients with c-ERβ only, n-ERβ only, and no ERβ expression patterns (4.7, 8.1, and 7.8 months, respectively), although statistical significance was not reached (*P *= 0.14).

Given the predictive value of EGFR sensitizing mutations in EGFR-TKI treatment, correlations between ERβ expression and survival after EGFR-TKIs in the subgroup with EGFR sensitizing mutations were analyzed. In patients with EGFR mutations (*n *= 107), cases with c-ERβ and n-ERβ co-expression (*n *= 14) had an inferior median PFS after EGFR-TKI treatment (4.1 months, 95%CI: 2.8 to 8.3 months) compared to patients without the same ERβ expression pattern (*n *= 93, PFS: 10 months, 95%CI: 8.4 to 11.6 months, *P *= 0.03) (Fig. 1C).

Several common EGFR-TKI resistance related gene variants (including mutations in KRAS, BRAF, PIK3CA and T790M, and c-MET amplification) were detected in patients who had EGFR mutations and c-ERβ and n-ERβ co-expression. Distribution of ERβ expression and genetic variants by case are listed in Table 2. After patients with genetic variants associated with EGFR-TKI resistance were removed, the median PFS in the remaining patients with c-ERβ and n-ERβ co-expression was 4.3 months (95%CI: 1.8 to 8.8 months).

### Intracellular ERβ localization was associated with ERβ isoforms

Previous studies have reported that intracellular ERβ localization (c-ERβ or n-ERβ) was due to different expression pattern of ERβ isoforms in some cancers. In lung cancers, ERβ isoforms 1, 2 and 5 are commonly expressed^13^. To check the localization of ERβ isoforms, we fused EGFP to the cDNA of these different isoforms of ERβ (1, 2 or 5) and transiently transfected them into PC9 and HeLa cells. As shown in Fig. 1D, ERβ isoform 1 (ERβ1) mainly localized in the nucleus both in transfected PC9 and HeLa cells, while ERβ isoforms 2 and 5 (ERβ2 and ERβ5) majored localized in the cytoplasm.

### Interactions between ERβ isoform 1 and other isoforms conferred resistance to gefitinib *in vitro*

To figure out the mechanism underlying the resistance to gefitinib, *In vitro* experiments were performed to identify whether c-ERβ and n-ERβ co-expression was a predicting factor associated with resistance to EGFR-TKI observed in clinical analyses.

**Figure 2:**
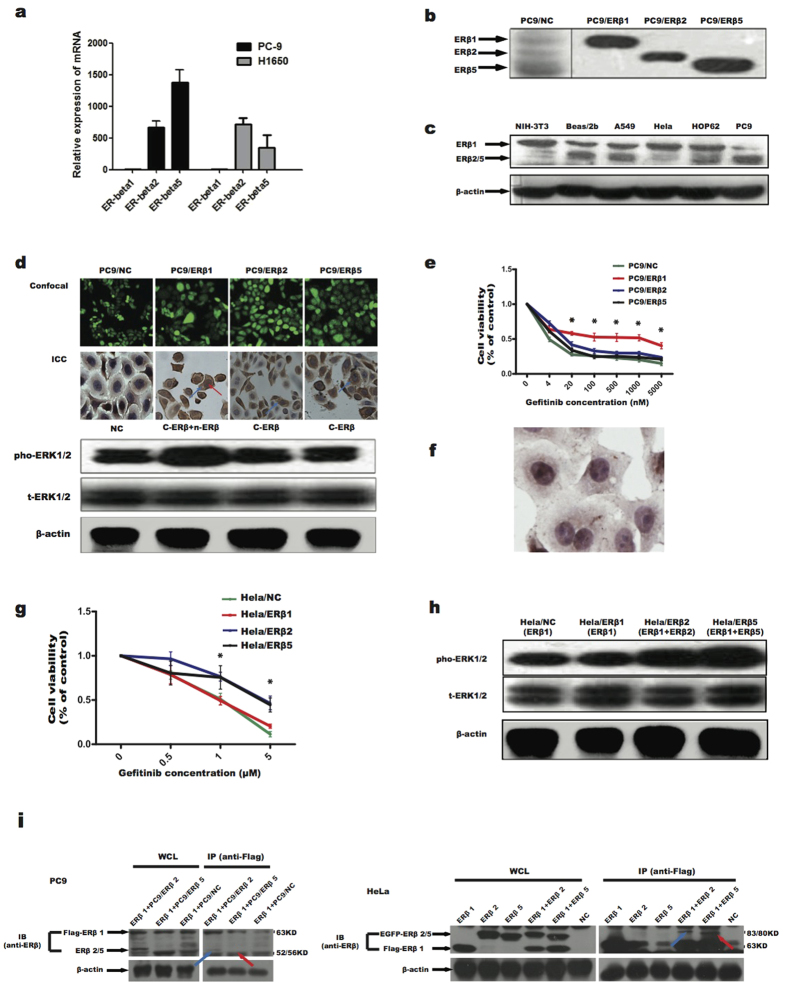
Co-expression of c- ERβ and n- ERβ conferred resistance to gefitinib due to the interactions between different ERβ isoforms. (**A**) Real-time PCR test showed both PC9 cells and H1650 cells mainly carried ERβ2 and ERβ5 but not ERβ1 in RNA levels. (**B**) Immunoblotting tests by anti-ERβ antibody showed that PC9 cells mainly harbored ERβ5 (left), PC9 cells transfected with ERβ1, ERβ2 or ERβ5 as positive control (right). Data were representative of two independent experiments. (**C**) Immunoblotting tests by anti-ERβ antibody showed that different expression pattern of intracellular ERβ in distinct cell lines. (**D**) Confocal imagings confirmed that PC9 cells were stably transfected with negative control vector (NC) and different ERβ isoforms (ERβ1, ERβ2 and ERβ5), which corresponded to different ERβ expression patterns in immunocytochemistry staining (ICC) (NC, c- ERβ+n- ERβ, c- ERβ and c- ERβ respectively) (c- ERβ, blue arrow; n- ERβ, red arrow). Immunoblotting tests illustrated that only PC9 cells with c- ERβ+n- ERβ showed enhanced pERK1/2 compared with NC. Data were representative of two independent experiments of each cell lines. (**E**) Cell viability test for 72 hours showed that PC9/ERβ1 cells were more resistant to gefitinib compared with PC9/ ERβ2 and PC9/ ERβ5 cells (* *p *< 0.05=. (**F**) HeLa cells mainly carried n- ERβ expression in ICC test. (**G**) Cell viability test for 72 hours showed that HeLa/ERβ1 cells were less resistant to gefitinib compared with HeLa/ERβ2 and HeLa/ERβ5 cells (* *p* < 0.05=. (**H**) HeLa/ERβ2 or 5 but not HeLa/ERβ1 cells showed enhanced activation of pERK1/2 in the immunoblotting test. Data were representative of two independent experiments of each cell lines. (**I**) WCLs and IP of indicated cell lines were used for immunoblotting with anti-ERβ and anti-Flag respectively. Both ERβ2 (blue arrow) and ERβ5 (red arrow) were observed coexisting with ERβ1 in IP tests. In PC9 cells (left), PC9 related cells (PC9/NC, PC9/ ERβ2 and PC9/ ERβ5 cells) were stably transfected with Flag-ERβ1. In HeLa cells (right), HeLa cells were transiently transfected with different plasmids (Flag-ERβ1, EGFP-ERβ2, EGFP-ERβ5, Flag-ERβ1+EGFP-ERβ2, Flag-ERβ1+EGFP-ERβ5 and NC).

As shown by real-time PCR and immunoblotting tests, PC9, a lung adenocarcinoma cell line with the EGFR 19del, expressed both ERβ isoforms 2 and 5 (Fig. 2A–C). To mimic clinical processes, we transfected ERβ 1, 2 or 5 plasmids into PC9 cells and constructed stable cell lines. PC9/ERβ1 cells (PC9 cell line with ERβ1) showed strong co-expression of c-ERβ and n-ERβ compared to PC9/NC cells (PC9 cell line with control vector), which was in contrast to PC9/ERβ2 and PC9/ERβ5 cells (PC9 cell line with ERβ2 or ERβ5) that only expressed c-ERβ (Fig. 2D). Cell viability tests indicated that PC9/ERβ1 cells had significant resistance to gefitinib compared with controls (PC9/NC) and the other two cell lines (PC9/ERβ2 and PC9/ERβ5) (Fig. 2E). Examination of the downstream signaling by immunoblotting test showed that the phosphorylated ERK1/2 was significantly enhanced in PC9/ERβ1, but not PC9/ERβ2 and PC9/ERβ5, compared with PC9/NC cells (Fig. 2D). Considering that PC9 cells mainly expressed ERβ2 and ERβ5, It seemed that only co-exist of ERβ1 and ERβ2 or ERβ1 and ERβ5 could induce the resistance, suggesting the possible role of an interaction between ERβ1 and other ERβ isoforms.

We further selected HeLa cells (primarily express n-ERβ as determined by immunoblotting test, Fig. 2C, and ICC tests, Fig. 2F) to identify the mechanism of PC9/ERβ1 cell resistance to gefitinib. ERβ1, 2 or 5 were transfected into HeLa cells stably, demonstrating that HeLa/ERβ1 cells showed a similar sensitivity as HeLa/NC cells, but were less resistant to gefitinib than HeLa/ERβ2 or HeLa/ERβ5 cells (Fig. 2G). The immunoblotting test showed that phosphorylated ERK1/2 was significantly enhanced in both HeLa/ERβ2 and HeLa/ERβ5, but not HeLa/ERβ1, compared to HeLa/NC cells (Fig. 2H).

To confirm the interactions between ERβ1 and ERβ2 or ERβ1 and ERβ5, the protein interaction tested by IP in PC9 and HeLa cells were performed (Fig. 2I). Lentivirus embedded Flag-ERβ1 plasmid was transfected to different cells (PC9/ERβ2 cells, PC9/ERβ5 cells and PC9/NC cells) under the treatment of gefitinib (1 μM). Whole cell lysates (WCLs) were used for immunoblotting with anti-ERβ. The IP tests were performed by anti-Flag. Both ERβ2 and ERβ5 were observed coexisting with ERβ1 in IP. Similarly, HeLa cells were transiently transfected with different plasmids respectively (Flag-ERβ1, EGFP-ERβ2, EGFP-ERβ5, Flag-ERβ1+EGFP-ERβ2 and Flag-ERβ1+EGFP-ERβ5) under the treatment of gefitinib and were used for IP with anti-Flag followed by immunoblotting with anti-ERβ. WCLs were used for immunoblotting with anti-ERβ. Initially when the mixed ratio between Flag-ERβ1 and EGFP-ERβ2 or EGFP-ERβ5 was 1:2, both ERβ2 and ERβ5 bands were observed coexisting with ERβ1 in IP, which suggested the interactions between ERβ1 and other isoforms.

Taken together, all these data demonstrated that co-expression of n- ERβ1 and c-ERβ conferred the resistance of NSCLC to EGFR-TKI treatment, which was due to the interactions between ERβ1 and ERβ2 or ERβ1 and ERβ5.

### Activation of intracellular non-genomic pathways mediated gefitinib resistance

**Figure 3:**
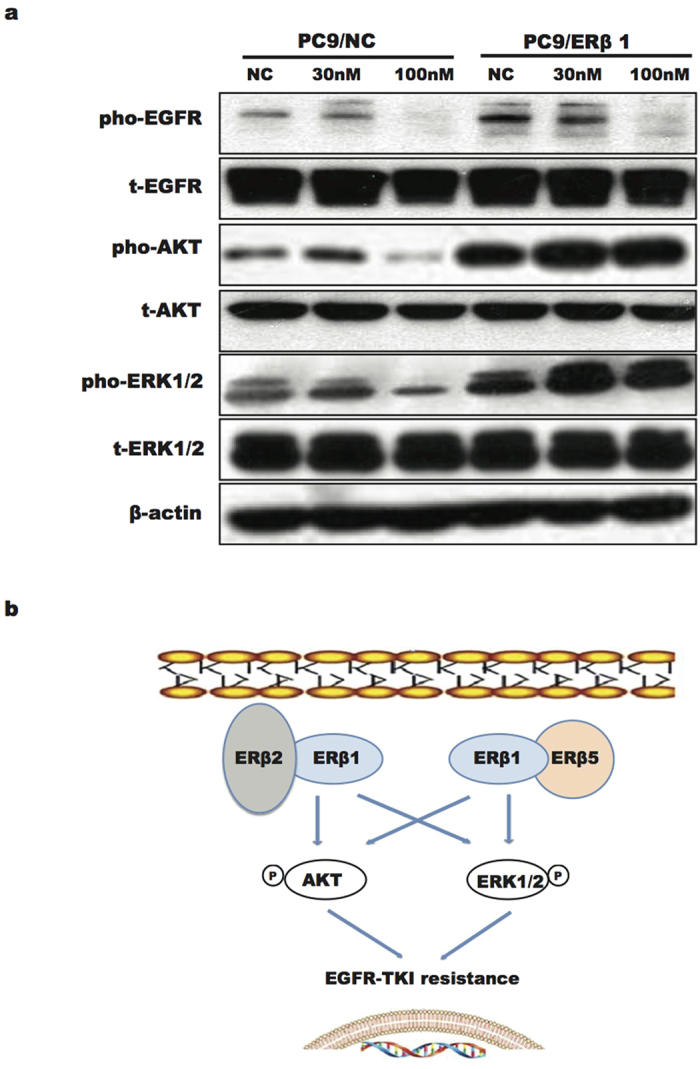
The activations of non-genomic signaling pathways mediated the resistance to gefitinib. (**A**) Cells were lysed for immunoblotting test after treated with gefitinib (vehicle, 30 nM, 100 nM) and E2 (10 nM) for 24 hours. Non-genomic signaling pathways including PI3K-AKT and ERK were significantly activated in PC9/ERβ1 cells under the treatment of gefitinib (100 nM) compared to PC9/NC cells. Data were representative of two independent experiments for immunoblotting tests of each concentrations of gefitinib. (**B**) The diagram showed the mechanism of EGFR-TKI resistance.

We further examined activation of intracellular non-genomic signaling pathways in PC9/NC and PC9/ERβ1 cells treated with gefitinib. Under various concentrations (vehicle, 30 nM and 100 nM), phosphorylation of ERK1/2 and AKT was increased in PC9/ERβ1 cells compared with the attenuated status of PC9/NC cells when treated with 100 nM gefitinib (Fig. 3A). Together with these data and the interactions between different ERβ isoforms identified above, a diagram was fabricated (Fig. 3B).

### Fulvestrant improved sensitivity to EGFR-TKI therapy in PC9/ERβ1 cells and patients with EGFR mutations and c-ERβ and n-ERβ co-expression

**Figure 4:**
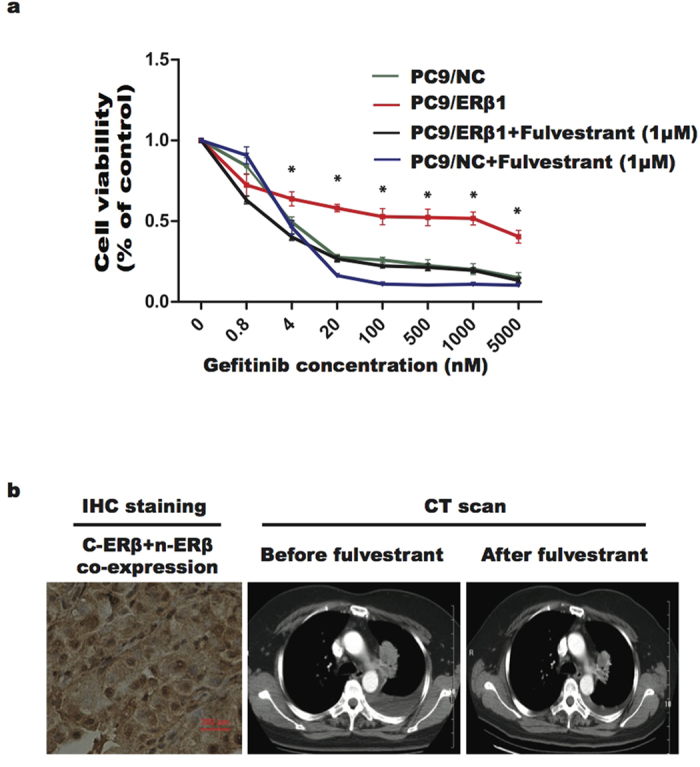
ER antagonist (fulvestrant) improved the sensitivity to gefitinib in PC9/ERβ1 Cells and EGFR mutant patients with c-ERβ and n-ERβ co-expression. (**A**) Cell viability of 72 hours after combined gefitinib with or without fulvestrant showed that fulvestrant recovered the sensitivity to gefitinib of PC9/ERβ1 as similar with PC9/NC cells (* *p* < 0.05=. (**B**) Antitumor of combined gefitinib and fulvestrant in one patients with co-expression of c-ERβ and n-ERβ. A 57-year-old Chinese female with lung adenocarcinoma and EGFR exon 19del underwent PD in primary lung lesion after 8.7 months of gefitinib and then received continuous gefitinib and locally radiation. When rapid PD (primary lung lesion and bone metastasis) was observed, fulvestrant combined with gefitinib was delivered, and 3 months of prolonged PFS was obtained. (left) IHC of ERβ, (middle) CT scan imaging before fulvestrant, and (right) CT scan imaging after 1.5 months of fulvestrant.

Following combined treatment with fulvestrant (1 μM), PC9/ERβ1 cells became sensitized to gefitinib, similar to PC9/NC cells. Fulvestrant also enhanced the antitumor activity of gefitinib in PC9/NC cells, particularly at relatively high concentrations (Fig. 4A).

To confirm the role of fulvestrant in reversing resistance to EGFR-TKIs, we enrolled one female Chinese patient with stage IV lung adenocarcinoma and an EGFR mutation. This patient underwent local progression of a primary lung lesion after 8.7 months of gefitinib treatment, and then received continuous gefitinib therapy plus localized radiation. When rapid PD was observed (primary lung lesion and bone metastasis), gefitinib combined with fulvestrant was administered based on positive c- ERβ and n-ERβ expression in sample tissue. Subsequently, 3 months of disease control was observed. CT scans showed tumor shrinkage although it failed to achieve partial remission of disease (Fig. 4B).

## Discussion

As transmembrane proteins, ERs share similar intracellular non-genomic signaling pathways with EGFR, suggesting that activating ER pathways may cause resistance to EGFR-TKIs^6^,^8^,^9^. However, correlation of ER expression with EGFR-TKI efficacy remains controversial. In the present study, c-ERβ and n-ERβ co-expression was identified as a potential biomarker for predicting poor PFS with EGFR-TKI therapy, which was examined as an outcome of the interaction between different ERβ isoforms. To the best of our knowledge, this represents the first study to correlate ERβ localization and resistance following EGFR-TKI treatment.

Based on initial clinical data, c-ERβ and n-ERβ co-expression (c-ERβ+n-ERβ) predicted inferior PFS after EGFR-TKI therapy compared to patients without this type of expression pattern. However, c-ERβ only patients also presented with a poor PFS. To identify the actual factors related to EGFR-TKI resistance, *in vitro* experiments mimicking clinical processes were performed. Through transfection with different ERβ isoforms, EGFR mutant lung cancer cells with c-ERβ and n-ERβ co-expression (PC9/ERβ1) or only c-ERβ expression (PC9/ERβ2 and PC9/ERβ5) were constructed as *in vitro* models. Significant resistance to gefitinib in PC9/ERβ1 cells compared with PC9/ERβ2 and PC9/ERβ5 cells supported the important effect of c-ERβ and n-ERβ co-expression. Therefore, c-ERβ and n-ERβ co-expression could be used as a biomarker predicting poor survival after EGFR-TKI therapy.

In the *in vitro* study, PC9 and HeLa cells were identified as expressing ERβ2/ERβ5 isoforms and the ERβ1 isoform, respectively. By transfecting different ERβ isoforms, various ERβ isoform combinations were constructed in PC9 (ERβ2/ERβ5+ERβ1, ERβ2/ERβ5+ERβ2, and ERβ2/ERβ5+ERβ5) and HeLa cells (ERβ1+ERβ1, ERβ1+ERβ2, and ERβ1+ERβ5). Only co-existence of ERβ1 and ERβ2 or ERβ1 and ERβ5 (namely, PC9 cells with ERβ2/ERβ5+ERβ1 and HeLa cells with ERβ1+ERβ2 or ERβ1+ERβ5) activated phosphorylated ERK1/2. Importantly, in the IP tests with anti-Flag-ERβ1, ERβ2 and ERβ5 were also pulled down. These results indicate that the interactions between ERβ1 and other isoforms induced gefitinib resistance. Given that commercially mature specific antibodies for different ERβ isoforms are not currently available^14^, c-ERβ and n-ERβ co-expression in IHC may represent concurrent ERβ1 and ERβ2 or ERβ5, which suggests that c-ERβ and n-ERβ co-expression might be a candidate biomarker for patient selection of primary resistance to EGFR-TKIs.

Consistent with previous reports^15^,^16^, we identified activation of intracellular pathways, such as PI3K-AKT-mTOR and MAPK, after the EGFR pathway was blocked, which indicated that the non-genomic signaling pathway mediated gefitinib resistance. Several studies have reported that ERβ2 and ERβ5 failed to form homodimers, but could heterodimerize with ERβ1 and enhance transactivation in a ligand-dependent manner^13^,^14^,^17^. We speculated that heterodimerization of ERβ1 and other isoforms activate non-genomic signaling pathways when cancer cells with both ERβ1 and other isoforms are treated with EGFR-TKI. A recent study from Nikolos’ team seemed to obtain the contradictory results which demonstrated that nuclear ERβ1 can down-regulate the EGFR and MAPK signaling pathway^18^. There are several differences between Nikolos’ and our study. First, we used different lung cancer cell line and we just focused on EGFR mutant lung cancer cells. However, Nikolos’ study did not show the effects of ERβ1 on lung cancer cells with EGFR mutation. Second, we herein explored the role of ERβ isoform interactions to gefitinib resistance. However, Nikolos’ study did not show the effect of ERβ1 after gefitinib delivery. Third, we illustrated that the activation of non-genomic signaling pathway by the interaction of ERβ isoforms in cytoplasm mediated the gefitinib resistance, which was different from nuclear ERβ1 affecting the transcription of target genes and then regulating the ERK1/2 signaling in Nikolos’ study. So, we think that ERβ1 can down-regulate EGFR and ERK1/2 signaling in NSCLC cells just like Nikolos’ study. However, after the treatment of EGFR-TKI, the interactions of ERβ isoforms will induce the EGFR-TKI resistance by activating non-genomic signaling pathway (such as ERK1/2 and AKT) especially in EGFR mutant lung cancer cells with co-expression of ERβ1 and other ERβ isoforms (ERβ2 or ERβ5).

To date, several genetic variants associated with EGFR-TKI resistance have been reported, such as the KRAS mutation^19^, PIK3CA mutation/amplification^20^, T790M mutation and c-MET amplification^21^,^22^. To exclude the effects of these factors associated with *de novo* resistance to EGFR-TKIs, several common variants were further analyzed in patients with EGFR mutations who also had c-ERβ and n-ERβ co-expression. Only 2 patients had the resistance-related gene mutations, which did not change the poor PFS after EGFR-TKI treatment in this population. These results supported the concept that c-ERβ and n-ERβ co-expression might be one of the mechanisms contributing to primary EGFR-TKI resistance.

Several *in vitro* and *in vivo* studies have shown enhanced effects when combining gefitinib and an ER inhibitor (e.g. tamoxifen or fulvestrant) in NSCLC, possibly providing a rationale for combining EGFR-TKIs with anti-estrogen therapy^10^,^11^. A pilot clinical study of combination therapy with gefitinib and fulvestrant in NSCLC also demonstrated improved anti-tumor activity^23^. In the present study, combined therapy consisting of gefitinib and fulvestrant led to enhanced anti-proliferative activity in EGFR-mutant lung cancer cells and improved PFS in adenocarcinoma patient with an EGFR mutation. Importantly, cell models and the one enrolled patient both had concurrent c-ERβ and n-ERβ expression, which provided a type of biomarker for alternative selection. However, only 3 months of prolonged PFS was observed for the selected patient when fulvestrant was added, which seemed to be inferior to the *in vitro* results. Possible reasons are that the timing of fulvestrant delivery was not appropriate in the patients. Initiating gefitinib combined with fulvestrant may be a more reasonable strategy for reversing EGFR-TKI resistance induced by concurrent c-ERβ and n-ERβ expression than combination therapy given after disease progression. Second, an insufficient dosage of fulvestrant may influence PFS improvement, and administration of fulvestrant twice rather than once per month is recommended in future clinical studies.

In summary, c-ERβ and n-ERβ co-expression predicted poor PFS after EGFR-TKI treatment in advanced NSCLC patients with an EGFR mutation. ERβ co-expression might serve as a candidate biomarker for predicting prognosis following EGFR-TKI therapy and determine if combined EGFR-TKI and ER inhibitor therapy is appropriate. The innate mechanism of resistance was activation of non-genomic signaling pathways mediated by interactions between ER-β1 and other isoforms. Further studies with larger samples to evaluate ERβ with EGFR-TKIs were warranted.

## Methods

### Patient selection

This study included 184 Chinese patients with advanced NSCLC who received an EGFR-TKI (gefitinib oral 250 mg/d or erlotinib oral 150 mg/d) at the Peking University Cancer Hospital between June 2005 and December 2013. All diagnoses were histologically proven and evaluated as stage IV according to the current TNM staging system (IASLC 2009). Only patients with sufficient tissue for both EGFR mutation analysis and ERβ immunohistochemistry staining were enrolled. One patient with cytoplasmic and nuclear ERβ co-expression was prospectively enrolled to receive combined fulvestrant therapy (250 mg, intramuscular injection once monthly) following disease progression after gefitinib treatment.

Specimens were stored according to protocols approved by the Institutional Review Board of Beijing Cancer Hospital, and informed consent to use biopsy tissues for sample analyses was obtained from all patients.

For all patients, medical records were reviewed to extract clinicopathological data. Responses were classified using standard Response Evaluation Criteria in Solid Tumors, version 1.1. PFS was assessed from the first day of EGFR-TKI treatment until radiologic progression or death. Overall survival (OS) was determined from the EGFR-TKI start date until the date of death. Patients without a known date of death were censored at the time of the last follow-up.

### Detection of EGFR sensitive and resistance related genetic variants

Genetic variants involved in this study included EGFR sensitizing mutations (exon 19del and 21L858R), EGFR T790M, PIK3CA, KRAS or BRAF mutations and c-MET amplification^1^,^23^,^24^,^25^,^26^. Briefly, EGFR sensitizing mutations were detected by denaturing high performance liquid chromatography (DHPLC) according to previously described methods. Amplification refractory mutation system (ARMS) was used to reevaluate the EGFR wild type patient with adenocarcinoma by DHPLC. Other mutations in EGFR T790M, PIK3CA, KRAS and BRAF were also detected by ARMS. C-MET amplification was determined by quantitative real-time PCR using the Stratagene Mx3000P Real-Time PCR System (Agilent Technologies, Santa Clara, CA, USA) with TaqMan Universal PCR Master Mix (Applied Biosystems, Foster City, CA, USA). The reference gene was RNasP, and the MET primer and probe were designed by Applied Biosystems (Hs01432482_cn). Normal human genomic DNA was used as a control. c-MET gene amplification was defined as: 2-ΔΔCT > 2.5 (ΔCT* *= CTMET – CTRNasp, ΔΔCT* *= ΔCTcase – ΔCTnormal).

### Immunohistochemistry and immunocytochemistry

ERβ expression was analyzed in lung tissue samples and cell lines using immunohistochemistry (IHC) and immunocytochemistry (ICC), respectively. Briefly, dried 4-micron slides with formalin-fixed, paraffin embedded tissue were prepared. Combined sodium citrate (pH 6.0) and incubation in a pressure cooker (3 min, 125 °C) was used for antigen retrieval. Slides were then incubated overnight at 4 °C with primary mouse monoclonal anti-human ERβ (ABCAM, UK) at a dilution of 1:100. A two-step polymer-HRP method (Dako, Carpinteria, CA) was used for detection. No staining was observed for negative controls, which included incubation of lung tissue with a non-immune primary antibody.

Immunoreactivity ‘positive’ of IHC was defined if more than 10% of cancer cells were stained. Based on the localization of ‘positive’ immunoreactivity in either the cytoplasm or nucleus, patients were grouped as either c-ER- and/or n-ER-positive.

IHC and ICC staining was evaluated independently by different investigators (Dr. Hua Bai and Dr. Xiaosheng Ding) and a pathologist (Yu Sun).

### Cell culture and chemicals

Human NSCLC cell lines (A549, HOP-62), HeLa cells, human bronchial epithelial cells (Beas/2b), mouse fibroblast cells (NIH-3T3) and human breast cancer cells (MCF-7) were provided by the National Institute of Biologic Sciences in Beijing. PC9 was a gift from the Guangdong Lung Cancer Institute. Gefitinib (EGFR-TKI), estradiol (E2) and fulvestrant (ER antagonist) were commercially obtained from Sigma-Aldrich. Agents (fulvestrant) administrated to patients were provided by AstraZeneca.

### Immunoblotting analysis

The protein expressions in cells were evaluated with western blot. Whole cell lysates (WCL) were obtained by extraction in cell lysis buffer (cell signaling) followed by protein quantification using the bicinchoninic acid assay (Pierce) and lysis in Laemmli sample buffer. A total of 20 ug of the protein sample was run on a 10% Tris-glycine gel and transferred to nitrocellulose. Primary antibodies were added and incubated overnight at 4 °C, and secondary antibodies were conjugated to horseradish peroxidase for 2 hours at room temperature. Blots were developed by enhanced chemiluminescence and photographed using a Fujifilm Dark Box II and Image Reader LAS-1000 Plus software. Primary antibodies included ERβ, EGFR, pEGFR, ERK1/2, pERK1/2, AKT, pAKT and β-actin (Santa Cruz). Peroxidase labeled anti-rabbit or anti-mouse secondary antibodies (Amersham Pharmacia, Piscataway, NJ) were used.

### Construction of pEGFP-ERβ isoform1, ERβ isoform 2 or ERβ isoform 5 and transient transfection

Localization of different ERβ isoforms was evaluated through transient transfection of ERβ isoform1 (ERβ1), isoform2 (ERβ2) or isoform5 (ERβ5). ERβ isoform 1, 2 and 5 fragments were synthesized by Genepharma (Shanghai, China). pEGFP-C1 vector was a kind gift from Dr. Xiaodong Wang (National Institute of Biological Sciences, Beijing). Briefly, pEGFP-C1 was digested with HindIII and BamHI. ERβ isoform fragments were amplified using PCR and ligated into HindIII and BamHI sites of pEGFR-C1. For transient transfection, cells were seeded at a density of 2 × 105 in 6-well plates overnight. A mixture of 1 μg plasmid and 3 μl lipofectamine was prepared in opti-MEM according to the manufacturer’s instructions and added to the cells. Forty-eight hours after transfection, ERβ-green fluorescent protein fusion was detected under a fluorescent microscope. Localization of various ERβ isoforms was determined by confocal imaging which was performed using a laser scanning LSM 510 confocal microscope (Carl Zeiss, Welwyn Garden City, UK).

### Protein interactions by immunoprecipitation (IP)

Flag-ERβ1 plasmid was modified from constructed EGFP-ERβ1 plasmid. HeLa cells were transiently transfected with different plasmids separately (Flag-ERβ1, EGFP-ERβ2, EGFP-ERβ5, Flag-ERβ1+EGFP-ERβ2 and Flag-ERβ1+EGFP-ERβ5). The transfection was performed with 2 μl lipo2000 (Invitrogen) per microgram plasmid. After 36 hours of transfection, cells were washed twice with ice-cold phosphate-buffered saline (PBS) and lysed in ice-cold lysis buffer. Bicinchoninic acid (BCA) protein assay kit (Beyotime) was used to measure the protein concentration. Equal amount of protein was immunoprecipitated with the Anti-Flag M2-Agarose from mouse (Sigma) and then subjected to 8% SDS–polyacrylamide gel electrophoresis (SDS-PAGE) The protein was then transferred from the gels onto polyvinylidene fluoride (PVDF) membranes and the immunoblotting to ERβ was performed as above described.

### Construction of stable cell lines using lentivirus transduction

ERβ1 (including ERβ1 and Flag-ERβ1), ERβ2 and ERβ5 were constructed into a lentivirus expression vector and packaged by Genepharma (Shanghai, China). Virus titers of the supernatants, including virus particles provided, ranged from 5 × 107 to 2 × 108. MOI of 50 were used for infection of PC9 cell lines. After 3 days of infection, 2 μg/ml puromycin was added to the cells and a stably pooled population of cells was obtained after 5 days. Stable integration of ERβ was determined by western blot.

### RNA extraction and real-time PCR

Total RNA was extracted using Trizol reagent according to the manufacturer’s instructions. First-strand cDNA synthesis was performed using superscript reverse transcriptase (Tiangen, Beijing). Relative mRNA expression levels of ER-beta isoforms were measured using the SYBR green assay (Toyobo, Japan). The sequence of primers used in RT-PCR was as follows: ER-beta1 forward primer 5'-GTCAGGCATGCGAGTAACAA-3', reverse primer 5'-GGGAGCCCTCTTTGCTTTTA-3’; ER-beta2 forward primer 5'-TCTCCTCCCAGCAGCAATCC-3', reverse primer 5'-GGTCACTGCTCCATCGTTGC-3'; ER-beta5 forward primer 5'-GATGCTTTGGTTTGGGTGAT-3', reverse primer 5'-CCTCCGTGGAGCACATAATC-3'; and GAPDH forward primer 5'-GACCCCTTCATTGACCTCAAC-3', reverse primer 5’-CTTCTCCATGGTGGTGAAGA-3'. Cycle values were determined using the system and analysis software. Comparative gene expression analysis was performed by normalizing to the level of GAPDH.

### Cell viability test

Cell viability was determined using a cell counting kit-8 (CCK8) (Dojindo, Japan). Briefly, cells were seeded in sextuplicate in 96-well plates containing 100 μl medium at a density of 2 × 103 cells/well for 24 hours and cultured with increasing concentrations of indicated drugs for an additional 72 hours. Afterward, 10 μl water soluble tetrazolium salt (WST-8) was added to each well and incubated for 3 hours. Absorbance was measured at 450 nm using a microplate reader. Relative viability was calculated as (%/control) = [A450 (treated) - A450 (blank)]/[A450 (control) - A450 (blank)].

### Statistics

Relationships between clinicopathologic factors were analyzed using Pearson’s χ2 test or Fisher’s exact test. Survival time was calculated using the Kaplan-Meier method, and comparisons between groups were made using log-rank tests. All statistical tests were two-tailed, with significance defined as *P* value less than 0.05. All analyses were performed using SPSS for Windows, version 17.0 (Statistical Package for the Social Sciences, Chicago, IL, USA).

## Additional Information

**How to cite this article**: Wang, Z. *et al.* ERβ localization influenced outcomes of EGFR-TKI treatment in NSCLC patients with EGFR mutations. *Sci. Rep.* **5**, 11392; doi: 10.1038/srep11392 (2015).

## Figures and Tables

**Table 1:** Clinical and pathological characteristics of 184 patients with advanced NSCLC.

**Variables**	**Number of cases (%)**
Age, years	
median	62
range	31-81
Gender	
Male	89 (48.4)
Female	95 (51.6)
Histology	
Adenocarcinoma	159 (86.4)
Non-adenocarcinoma	24 (13.6)
Smoking status	
Ever or current	54 (33.7)
Never or light*	122 (66.3)
EGFR mutation status	
Mutant type	114 (62.0)
Wild type	70 (38.0)
ERβ expression	
Positive	49 (26.6)
Negative	135 (73.4)
ERβ localization	
Nuclear only	22 (12.0)
Cytoplasmic only	5 (2.7)
Cytoplasmic + Nuclear	22 (12.0)

^*^Light smoking defined as smoking <100 cigarettes in lifetime.

**Table 2:** The gene aberrances and PFS after EGFR-TKI in patients with c-ERβ and n- ERβ co-expression.

**Case NO.**	**ERβ expression**	**EGFR mutation status**	EGFR-TKI resistance related gene aberrance*	**PFS after EGFR-TKI (months)**
1	c- + n-ERβ	21L858R	—	1.4
2	c- + n-ERβ	19del	—	4.3
3	c- + n-ERβ	19del	—	6.1
4	c- + n-ERβ	21L858R	—	22.1
5	c- + n-ERβ	19del	—	21.5
6	c- + n-ERβ	21L858R	—	18.2
7	c- + n-ERβ	21L858R	—	3.0
8	c- + n-ERβ	19del	KRAS+	3.1
9	c- + n-ERβ	21L858R	T790M+	13.7
10	c- + n-ERβ	21L858R	—	4.1
11	c- + n-ERβ	21L858R	—	1.2
12	c- + n-ERβ	19del	—	2.5
13	c- + n-ERβ	19del	—	8.6
14	c- + n-ERβ	21L858R	—	2.8

^*^EGFR-TKI resistance related gene variations including mutations in KRAS, B-raf, PIK3CA and T790M, and amplification in c-MET.

## References

[b1] Mok T. *et al.* Gefitinib or carboplatin-paclitaxel in pulmonary adenocarcinoma. N Engl J Med 361, 947–957 (2009). 19692680 10.1056/NEJMoa0810699

[b2] Maemondo M. *et al.* Gefitinib or chemotherapy for non-small-cell lung cancer with mutated EGFR. N Engl J Med 362, 2380–2388 (2010). 20573926 10.1056/NEJMoa0909530

[b3] Zhou C. C. *et al.* Gefitinib versus chemotherapy as first-line treatment for patients with advanced EGFR mutation-positive non-small-cell lung cancer (OPTIMAL, CTONG-0802): a multicentre, open-label, randomised, phase 3 study. Lancet Oncol 12, 735–742 (2011). 21783417 10.1016/S1470-2045(11)70184-X

[b4] Rosell R. *et al.* Gefitinib versus standard chemotherapy as fi rst-line treatment for European patients with advanced EGFR mutation-positive non-small-cell lung cancer (EURTAC): a multicentre, open-label, randomised phase 3 trial. Lancet Oncol 13, 239–246 (2012). 22285168 10.1016/S1470-2045(11)70393-X

[b5] Mitsudomi T. *et al.* Gefitinib versus cisplatin plus docetaxel in patients with non-small-cell lung cancer harbouring mutations of the epidermal growth factor receptor (WJTOG3405): an open label, randomised phase 3 trial. Lancet Oncol 11, 121–128 (2010). 20022809 10.1016/S1470-2045(09)70364-X

[b6] Enmark E. *et al.* Human estrogen receptor β-gene structure, chromosomal localization, and expression pattern. J Clin Endocrinol Metab 82, 4258–4265 (1997). 9398750 10.1210/jcem.82.12.4470

[b7] Omoto Y. *et al.* Expression, function, and clinical implications of the estrogen receptor beta in human lung cancers. Biochem Biophys Res Commun 285, 340–347 (2001). 11444848 10.1006/bbrc.2001.5158

[b8] Siegfried J. M., Hershberger P. A. & Stabile L. P. Estrogen receptor signaling in lung cancer. Semin Oncol 36, 524–531 (2009). 19995644 10.1053/j.seminoncol.2009.10.004 PMC2818861

[b9] Zhang G. *et al.* Estrogen receptor beta functions through nongenomic mechanisms in lung cancer cells. Mol Endocrinol 23, 146–156 (2009). 19106194 10.1210/me.2008-0431 PMC2818690

[b10] Stabile L. P. *et al.* Combined targeting of the estrogen receptor and the epidermal growth factor receptor in non-small cell lung cancer shows enhanced antiproliferative effects. Cancer Res 65, 1459–1470 (2005). 15735034 10.1158/0008-5472.CAN-04-1872

[b11] Siegfried J. M., Gubish C. T. & Rothstein M. E. *et al.* Combining the multitargeted tyrosine kinase inhibitor vandetanib with the antiestrogen fulvestrant enhances its antitumor effect in non-small cell lung cancer. J Thorac Oncol 7, 485–495 (2012). 22258476 10.1097/JTO.0b013e31824177ea PMC3288546

[b12] Nose N., Uramoto H. & Iwata T. *et al.* Expression of estrogen receptor beta predicts a clinical response and longer progression-free survival after treatment with EGFR-TKI for adenocarcinoma of the lung. Lung Cancer 71, 350–355 (2011). 20615575 10.1016/j.lungcan.2010.06.009

[b13] Leung Y. K., Mak P. & Hassan S. *et al.* Estrogen receptor (ER)-beta isoforms: a key to understanding ER-beta signaling. Proc Natl Acad Sci USA 103, 13162–13167 (2006). 16938840 10.1073/pnas.0605676103 PMC1552044

[b14] Leung Y. K. *et al.* Estrogen receptor beta2 and beta5 are associated with poor prognosis in prostate cancer, and promote cancer cell migration and invasion. Endocr Relat Cancer 17, 675–689 (2010). 20501637 10.1677/ERC-09-0294 PMC2891483

[b15] Verma M. K., Miki Y. & Abe K. *et al.* Cytoplasmic estrogen receptor β as a potential marker in human non-small cell lung carcinoma. Expert Opin Ther Targets 16, Suppl 1, S91–102 (2012). 22313325 10.1517/14728222.2011.630664

[b16] Pinton G. *et al.* Estrogen receptor β exerts tumor repressive functions in human malignant pleural mesothelioma via EGFR inactivation and affects response to gefitinib. PLoS One 5, e14110 (2010). 21124760 10.1371/journal.pone.0014110 PMC2993924

[b17] Dey P. *et al.* Estrogen receptors β1 and β2 have opposing roles in regulating proliferation and bone metastasis genes in the prostate cancer cell line PC3. Mol Endocrinol 26, 1991–2003 (2012). 23028063 10.1210/me.2012.1227 PMC3517717

[b18] Nikolos F., Thomas C. & Rajapaksa G. *et al.* ERβ Regulates NSCLC Phenotypes by Controlling Oncogenic RAS Signaling. Mol Cancer Res 12, 843–854 (2014). 24618619 10.1158/1541-7786.MCR-13-0663

[b19] Wang S. *et al.* Potential clinical significance of a plasma-based KRAS mutation analysis in patients with advanced NSCLC. Clin Cancer Res 16, 1324–1330 (2010). 20145159 10.1158/1078-0432.CCR-09-2672

[b20] Sequist L. V. *et al.* Genotypic and histological evolution of lung cancers acquiring resistance to EGFR inhibitors. Science Translational Medicine 75, 75ra26 (2011). 10.1126/scitranslmed.3002003 PMC3132801 21430269

[b21] Kobayashi S. *et al.* EGFR mutation and resistance of non-small-cell lung cancer to gefitinib. N Engl J Med 352, 786–792 (2005). 15728811 10.1056/NEJMoa044238

[b22] Pao W. *et al.* Acquired resistance of lung adenocarcinomas to gefitinib or gefitinib is associated with a second mutation in the EGFR kinase domain. PLoS Med 2, e73 (2005). 15737014 10.1371/journal.pmed.0020073 PMC549606

[b23] Traynor A. M. *et al.* Pilot study of gefitinib and fulvestrant in the treatment of post-menopausal women with advanced non-small cell lung cancer. Lung Cancer 64, 51–59 (2009). 18701186 10.1016/j.lungcan.2008.07.002 PMC3164240

[b24] Bai H. *et al.* Epidermal growth factor receptor mutations in plasma DNA samples predict tumor response in Chinese patients with stages IIIB to IV non–small-cell lung cancer. J Clin Oncol 27, 2653–2659 (2009). 19414683 10.1200/JCO.2008.17.3930

[b25] Newton C. R. *et al.* Analysis of any point mutation in DNA: the Amplification Refractory Mutation System (ARMS). Nucleic Acids Res 17, 2503–2516 (1989). 2785681 10.1093/nar/17.7.2503 PMC317639

[b26] Whitcombe D., Theaker J. & Guy S. P. *et al.* Detection of PCR products using self-probing amplicons and fluorescence. Nat Biotechnol 17, 804–807 (1999). 10429248 10.1038/11751

